# Genome-Scale Metabolic Modeling Combined with Transcriptome Profiling Provides Mechanistic Understanding of Streptococcus thermophilus CH8 Metabolism

**DOI:** 10.1128/aem.00780-22

**Published:** 2022-08-04

**Authors:** Martin H. Rau, Paula Gaspar, Maiken Lund Jensen, Asger Geppel, Ana Rute Neves, Ahmad A. Zeidan

**Affiliations:** a Systems Biology, R&D Discovery, Chr. Hansen A/S, Hørsholm, Denmark; b Biochemical Assays, Global Assay Development, Chr. Hansen A/S, Hørsholm, Denmark; University of Naples Federico II

**Keywords:** food fermentation, genome-scale metabolic modeling, microbial biotechnology, microbial metabolism, RNA-Seq, *Streptococcus thermophilus*

## Abstract

Streptococcus thermophilus is a lactic acid bacterium adapted toward growth in milk and is a vital component of starter cultures for milk fermentation. Here, we combine genome-scale metabolic modeling and transcriptome profiling to obtain novel metabolic insights into this bacterium. Notably, a refined genome-scale metabolic model (GEM) accurately representing S. thermophilus CH8 metabolism was developed. Modeling the utilization of casein as a nitrogen source revealed an imbalance in amino acid supply and demand, resulting in growth limitation due to the scarcity of specific amino acids, in particular sulfur amino acids. Growth experiments in milk corroborated this finding. A subtle interdependency of the redox balance and the secretion levels of the key metabolites lactate, formate, acetoin, and acetaldehyde was furthermore identified with the modeling approach, providing a mechanistic understanding of the factors governing the secretion product profile. As a potential effect of high expression of arginine biosynthesis genes, a moderate secretion of ornithine was observed experimentally, augmenting the proposed hypothesis of ornithine/putrescine exchange as part of the protocooperative interaction between S. thermophilus and Lactobacillus delbrueckii subsp. *bulgaricus* in yogurt. This study provides a foundation for future community modeling of food fermentations and rational development of starter strains with improved functionality.

**IMPORTANCE** Streptococcus thermophilus is one the main organisms involved in the fermentation of milk and, increasingly, also in the fermentation of plant-based foods. The construction of a functional high-quality genome-scale metabolic model, in conjunction with in-depth transcriptome profiling with a focus on metabolism, provides a valuable resource for the improved understanding of S. thermophilus physiology. An example is the model-based prediction of the most significant route of synthesis for the characteristic yogurt flavor compound acetaldehyde and identification of metabolic principles governing the synthesis of other flavor compounds. Moreover, the systematic assessment of amino acid supply and demand during growth in milk provides insights into the key challenges related to nitrogen metabolism that is imposed on S. thermophilus and any other organism associated with the milk niche.

## INTRODUCTION

Streptococcus thermophilus is a lactic acid bacterium with major industrial significance in the production of fermented dairy products, such as yogurt and cheese, and more recently also in the production of plant-based fermented foods. The main contribution of S. thermophilus in food fermentation is fast acidification through the production of lactic acid, in addition to texture and flavor formation (1). Natural niches of S. thermophilus include bovine milk and the mammary mucosa. Although it has been employed in milk fermentation for millennia, S. thermophilus was first isolated and described in 1919 (2). The organism is a facultatively anaerobic, Gram-positive, homofermentative lactic acid bacterium with a low G+C genomic content and a genome size around 1.8 to 2.0 Mb (3).

In evolutionary terms, S. thermophilus has in recent times (3,000 to 30,000 years) undergone adaptation to milk as a new environmental niche (3). As a homofermentative organism, S. thermophilus converts lactose into lactic acid, resulting in acidification, which is a hallmark of most milk fermentations. Other minor fermentation products include formate, acetaldehyde, pyruvate, acetoin, and ethanol (1, 4–6), which may function as flavor components. Apart from having an abundant carbon source, i.e., lactose, milk is also rich in nitrogen; however, it is mostly protein bound, requiring efficient proteolysis for feasible uptake and utilization. Some S. thermophilus strains harbor the gene encoding the extracellular serine endopeptidase PrtS, usually providing a growth advantage (7), and yet many strains are *prtS* negative (8). As a consequence, the main mechanism of amino acid acquisition in S. thermophilus is through peptide uptake and subsequent hydrolysis. Amino acid biosynthesis is also known to play an important role for growth of S. thermophilus in milk, especially the synthesis of sulfur and branched-chain amino acids (BCAAs) (9, 10). A frequent concern during milk fermentation is the risk of phage infection, and several phage types to which S. thermophilus is susceptible have been identified (11–13).

A few global microarray-based transcriptome studies have been conducted in S. thermophilus, with a focus on growth in milk (9), interactions with Lactobacillus delbrueckii subsp. *bulgaricus* (14, 15), bacteriophage infection (16), or heat shock (17), whereas transcriptome sequencing (RNA-Seq)-based studies have emerged recently focusing on exocellular polysaccharide (EPS) synthesis (18, 19); pH-controlled fermentation (20); and the effect of pH, temperature, and peptide composition (21, 22). Besides these system-wide studies, a strain-specific genome-scale metabolic model (GEM) has been developed for S. thermophilus LMG18311 (5), although it is accompanied only by a limited number of simulations and lacks a predicted nutrient uptake and product secretion profile. The availability of high-quality GEMs is known to enable a mechanistic system-level understanding of metabolism and offer a useful tool for guiding the development of strains and cultures with enhanced characteristics for food fermentations (23).

To qualify as yogurt, according to the FAO/WHO Codex Alimentarius, milk fermentation is required to be carried out by S. thermophilus in combination with Lactobacillus delbrueckii subsp. *bulgaricus* (24). In milk, the two species coexist presumably in a mutualistic relationship exchanging metabolites, e.g., formate, folate, ammonia, ornithine, putrescine, and pyruvate, that augment the growth of each other (14, 15, 25). Both organisms are classified as generally recognized as safe (GRAS), and S. thermophilus is devoid of the virulence factors present in other species within the Streptococcus genus. While the coculture of the two organisms is the typical mode of fermentation in an applied setting, studying S. thermophilus in isolation also has clear merit, as this organism by itself can impart most, if not all, the characteristics required for, e.g., the production of yogurt.

This study aims at achieving a mechanistic understanding of key aspects of S. thermophilus physiology and metabolism through the combination of *in silico* and experimental approaches. The generation of a refined GEM capable of accurately simulating growth and metabolite production of an industrial S. thermophilus strain provided detailed insights into the metabolic flux distribution under different conditions. Flux balance analysis (FBA) was used to explain the variation in the secretion product profile in response to changing the nitrogen source in the medium. In addition, model simulations were employed to guide the analysis and interpretation of global and differential gene expression profiles of S. thermophilus CH8 in milk and in a chemically defined medium in a metabolic network context. Overall, the genome-wide phenotypic characteristics of S. thermophilus during growth in milk could be identified based on the obtained results.

## RESULTS

### Global genome and transcriptome characteristics of S. thermophilus CH8.

**FIG 1:**
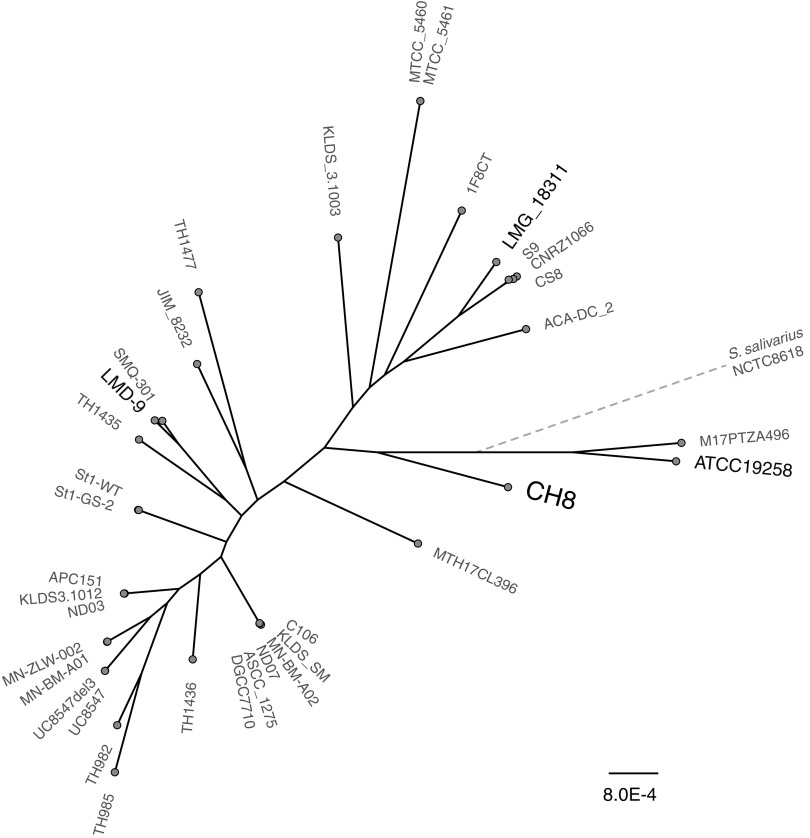
Maximum likelihood phylogeny of CH8 and certain S. thermophilus strains with publicly available genome sequences, as inferred from an alignment of 534 core genes. The location of *S. salivarius* strain NCTC8618 as an outgroup is indicated by a dotted line, which is not to scale.

S. thermophilus CH8 is a component of a commercial yogurt starter culture and is capable of efficient milk acidification, despite lacking the cell-envelope protease PrtS, as well as high texture formation. The genome of CH8 was sequenced to completion via a hybrid assembly approach, utilizing both short- and long-read sequencing technologies. The strain has a genome size of 1.85 Mb and is phylogenetically closely related to the type strain ATCC 19258, while it is separate from branches that include previously characterized strains, such as S. thermophilus LMD-9, LMG18311, and CNRZ1066 (Fig. 1). Based on rooting with Streptococcus salivarius NCTC8618, both CH8 and ATCC 19258 are relatively closer to the S. thermophilus ancestor. Nonetheless, clustering based on ortholog presence or absence places CH8 within a branch containing, e.g., LMG18311 and CNRZ1066 (see Fig. S1 in the supplemental material). Consequently, the genome sequence indicates a distinct ancestral origin, while the gene content resembles that of certain other previously characterized dairy strains. Comparing the ortholog distribution between CH8 and 34 previously published strains, with a focus on genes encoding metabolic functions, revealed only minor differences (see Table S1 in the supplemental material). Apart from specific genes in the exopolysaccharide synthesis gene cluster, CH8 also encodes an amino acid transporter that is rare among the other strains, while conversely lacking carbonic anhydrase, lysine decarboxylase (lysine degradation), and urocanate hydratase (histidine degradation) which are all present in the majority of the 34 strains. Therefore, at the level of metabolic genes, strain CH8 should provide a good representation of most strains within the species.

**FIG 2:**
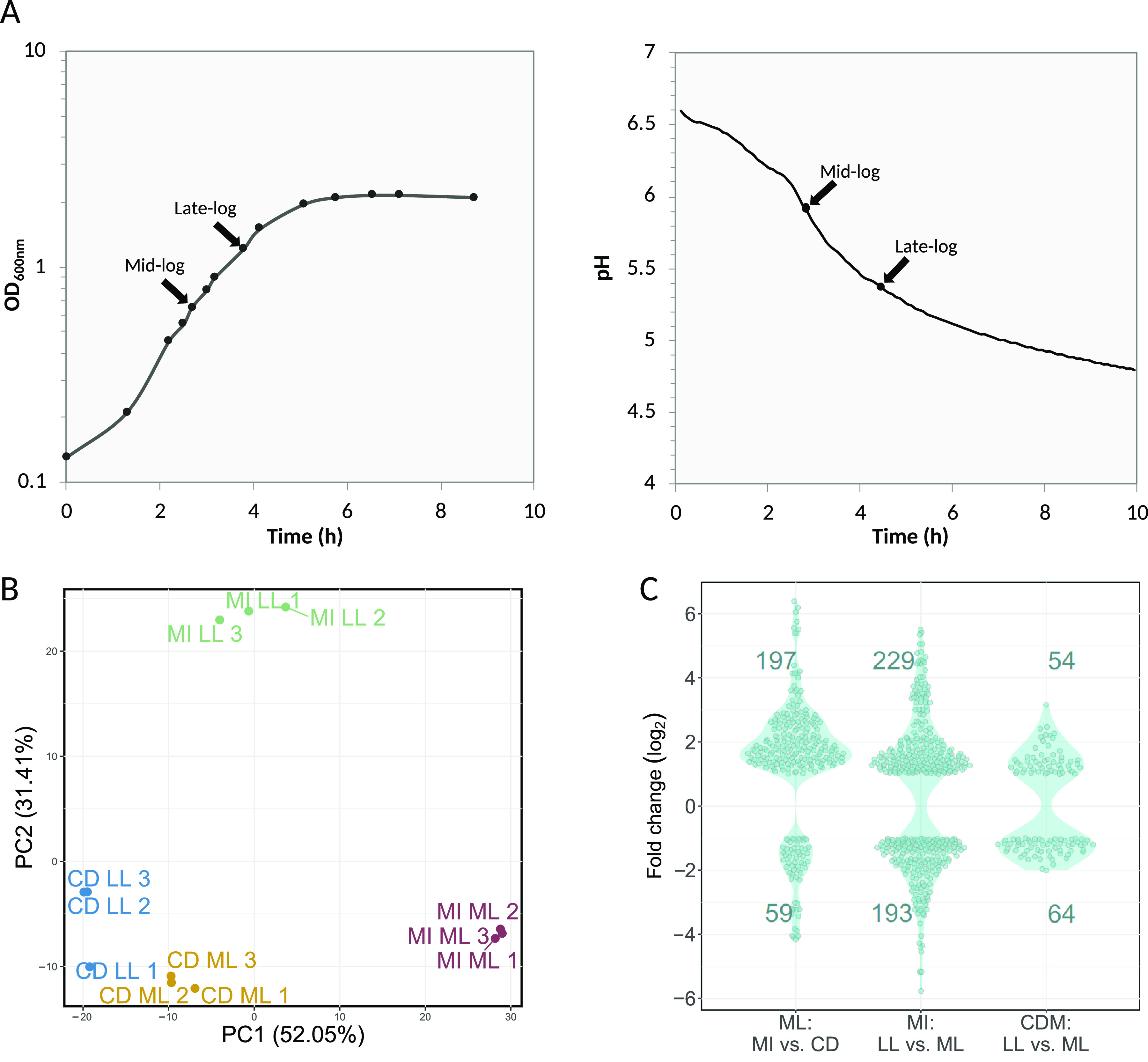
(A) Growth and acidification curves of CH8 in CDM (left) and milk (right). Arrows indicate OD_600_ and pH values of RNA sample harvest points. (B) Principal-component analysis plot of sample gene expression profiles. (C) Fold change distributions and quantity of differentially expressed genes, defined as having a *q* value below 0.05 and log_2_ fold change above |±1|. Expression changes are defined as values of the first condition in relation to values of the second condition. MI, milk; CD, CDM; ML, mid-log growth phase; LL, late-log growth phase.

To obtain an increased physiological knowledge on strain CH8 and S. thermophilus in general, the transcriptional response of the strain was assessed under different growth conditions using RNA-Seq. Two medium conditions were employed, namely, a chemically defined medium (CDM) (Fig. 2A, left panel) and milk (Fig. 2A, right panel). CDM is a nutrient-rich medium with defined components, including free amino acids. Two time points (1 and 2) were included in the analysis, corresponding to mid-log and late-log phases, with the second time point approaching the transition into the stationary phase of batch fermentation (Fig. 2A).

Replicate samples displayed similar transcription profiles, as indicated by the principal-component analysis (Fig. 2B). Here, mid-log and late-log samples from cultures in milk form clearly diverging groups, while CDM samples display moderate separation based on growth phase. Accordingly, medium composition, but also growth phase especially in milk, appears to have a profound effect on gene transcription. This finding can also be observed in the differential gene expression (Fig. 2C). While the quantity of differentially expressed genes is larger for the growth phase comparison in milk, the magnitude of fold change is slightly higher for the medium comparison.

### Expression of genes related to carbon metabolism.

**FIG 3:**
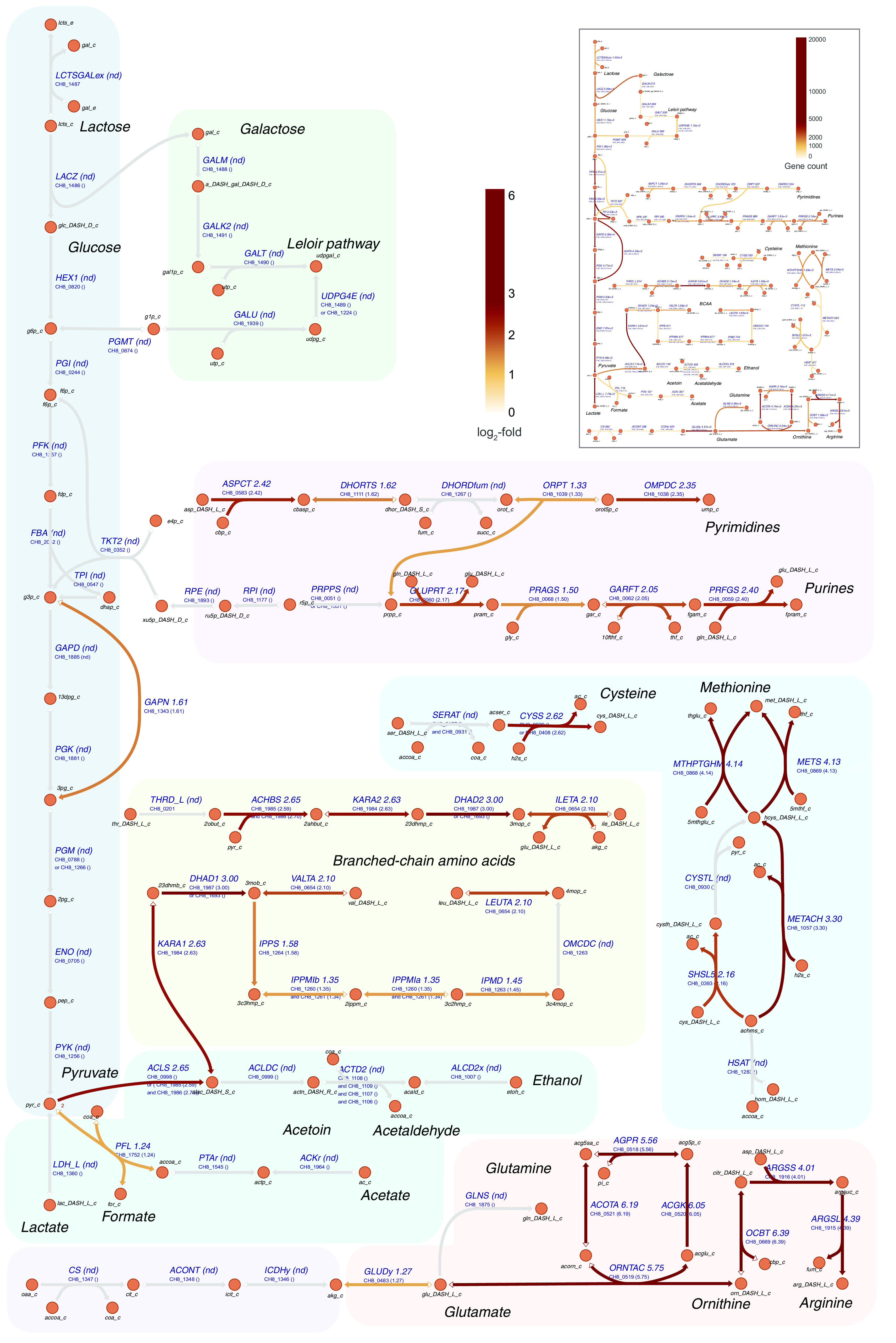
Map of CH8 metabolism overlaid with transcriptome data. The darker brown color signifies higher log_2_ fold expression changes between milk mid-log versus CDM mid-log conditions, while “nd” denotes reactions associated with genes displaying no statistically significant differential expression. Insert: transcription level of corresponding enzyme-encoding genes, as estimated from read counts. The darker color signifies higher read counts. For each gene, the highest gene count among conditions is selected.

Concerning specific gene expression changes (Fig. 3), genes involved in lactose metabolism, glycolysis, and the Leloir pathway displayed only minor fold changes (statistically significant, *q* value of <0.05, but below the log_2_ fold cutoff of >|±1|) between different conditions, except for *gapN*, encoding NADP-dependent glyceraldehyde-3-phosphate dehydrogenase. This gene displayed a 1.6 log_2_-fold higher expression in mid-log milk cultures than that in mid-log cultures in CDM and late-log cultures in milk, indicating a higher demand for NADPH, which is needed, for example, in amino acid and nucleotide biosynthesis. The *ldh* gene encoding lactate dehydrogenase follows the expression pattern of the glycolysis genes and also has a high expression level. Genes involved in the formation of other fermentation end products, such as formate, acetate, acetolactate, acetoin, acetaldehyde, and ethanol, displayed much lower expression levels; most are nonetheless above the median gene expression level. Genes involved in acetaldehyde synthesis (Fig. 3, insert) displayed disparate expression levels, from around 100 to 2,000 in milk mid-log phase, with acetolactate decarboxylase expression particularly low. The limited differential expression of pyruvate metabolism genes is observed, although pyruvate formate-lyase has more than double the expression in mid-log cultures in milk compared with mid-log cultures in CDM and late-log milk cultures; this result is likely a reflection of an increased nucleotide biosynthesis demand.

Genes related to the Leloir pathway generally displayed considerably lower expression levels compared with glycolytic genes (on average a log_2_ fold change of 4.2), indicating a much lower activity of the Leloir pathway, as expected. The Leloir pathway provides precursors for the biosynthesis of EPS whose biosynthesis genes were roughly halved in expression in late-log cultures, compared with mid-log cultures in both milk and CDM. As Leloir pathway genes contrarily displayed upregulation (*q* value of <0.05, but below the fold change cutoff), the divergence in the expression pattern of the Leloir pathway genes and EPS synthesis genes could indicate the involvement of different regulatory mechanisms in their expression.

### Expression of genes related to nitrogen metabolism.

Among genes related to nitrogen metabolism, more expression changes are observed than for those related to carbon metabolism. Numerous amino acid synthesis genes were upregulated in milk compared with those in CDM (Fig. 3; see Table S3 in the supplemental material). Most amino acid biosynthesis pathways included genes with a statistically significant log_2_ fold change above 1 in milk compared those in CDM, e.g., pathways for arginine, branched-chain amino acid (BCAA), cysteine, methionine, histidine, aspartate, glutamate, lysine, threonine, and aromatic amino acid biosynthesis. Glutamine and asparagine pathway genes were unchanged, whereas some genes involved in glycine and proline biosynthesis had slightly lower expression in milk (*q* value of <0.05, but below the fold change cutoff). In late-log cultures in milk, most amino acid synthesis genes had a somewhat lower expression than mid-log cultures, while tryptophan synthesis genes displayed markedly increased expression (2 to 4 log_2_ fold change). Regarding gene upregulation in milk compared with that in CDM, the fold changes of genes encoding enzymes in the pathway from glutamate to arginine are particularly striking with an average 5.5 log_2_-fold increase in milk (*q* value of <0.05). In mid-log cultures in milk, these genes reached very high read count levels, even comparable to the count levels of many glycolysis genes. Likewise, genes encoding enzymes involved in cysteine and methionine synthesis were upregulated markedly during growth under milk conditions.

Gene expression patterns also reflected the requirement for nucleotide biosynthesis in milk, as genes encoding enzymes involved in the synthesis of nucleotide precursors generally showed higher expression in mid-log cultures in milk than those in CDM, with a median log_2_ fold change of 2.3 (*q* value of <0.05).

Contrary to most amino acid synthesis genes, upregulation or differential expression of peptidase genes were generally limited, regardless of the variation in growth phase or medium composition. Possibly, peptidase gene regulation is limited and S. thermophilus may have evolved to express peptidase genes constitutively. Genome-wide differential expression and count values are provided in Table S3.

### Generation and refinement of a strain-specific genome-scale metabolic model.

The genome of CH8 provides a blueprint of its metabolic capabilities and forms the basis of the strain’s genome-scale metabolic model (GEM). By applying a comparative genomics approach, an S. thermophilus CH8-specific GEM, iRZ476, was created from previously published GEMs of five other organisms, including S. thermophilus LMG18311 (5, 26–30). Based on this approach, a matching ortholog could not be found in CH8 for only a few of the proteins included among the gene-protein-reaction associations in the LMG18311 model, and consequently, only two out of all 522 reactions were not transferred to the CH8 model. However, the comparison to the other reference models resulted in the incorporation of 50 additional reactions and improved the gene-protein-reaction association for 11 reactions. Through further manual curation based on knowledge of S. thermophilus physiology, pseudogene identification, and published literature (1, 14, 15), additional reactions were included or discarded, and further constraints were imposed on several key reactions originating from the LMG18311 model. Notably, reactions of pyruvate dehydrogenase, pyruvate oxidase, fumarate reductase, and alanine dehydrogenase were removed, while reactions of acetoin dehydrogenase and glyceraldehyde-3-phosphate dehydrogenase (GAPN) were added. These changes have profound effects on model simulations and more accurately reflect the metabolism of S. thermophilus as a species.

### Simulating growth in a chemically defined medium.

The refinement process led to a functional S. thermophilus GEM capable of predicting a realistic metabolite uptake and secretion profile, in concordance with previous experimental findings, e.g., the production of lactate, formate, acetoin, and succinate (5, 6, 14, 31). The CH8-specific GEM, therefore, has the potential to serve as an updated general S. thermophilus metabolic model and can be used to generate models specific to other members in the species with minimal effort. Including CH8-specific experimental data on substrate (lactose, galactose, and glucose) uptake and lactate secretion rates during growth in CDM as additional constraints generated a condition-specific model of high accuracy, as reflected in the predicted growth rate of 0.98 h^−1^, which is identical to the measured growth rate of the strain. The secretion products predicted by the model under these conditions include lactate, CO_2_, formate, acetaldehyde, fumarate, and glycerol (Table 1). The vast majority of CO_2_ synthesized is an effect of acetolactate metabolism and fatty acid synthesis, each responsible for ca. 50%, whereas formate is synthesized by pyruvate formate-lyase in the generation of acetyl-coenzyme A (CoA). The inclusion of experimental constraints led to predicting the secretion of acetaldehyde, an important yogurt flavor compound, at the expense of acetoin secretion, which is otherwise predicted in the absence of these constraints. The predicted production of acetaldehyde occurs via acetoin dehydrogenase activity as a means of NAD^+^/NADH redox balancing. A minor redox imbalance otherwise exists, as a consequence of a small excess of NAD^+^ regenerated through lactate dehydrogenase, in relation to the amount of NADH generated during glycolysis and the experimentally measured lactate secretion rate. Fumarate and glycerol secretion are also predicted, with or without experimental constraints. Fumarate is a by-product of nucleotide biosynthesis and subject to interconversion with succinate depending on redox balance, while glycerol is a by-product of cardiolipin biosynthesis. Despite being energetically favorable in theory, no acetate production is predicted when applying the described experimental constraints, which is also in agreement with acetate measurements (data not shown). Flux values for all reactions are provided in Table S4 in the supplemental material.

**TABLE 1:** Predicted uptake and secretion fluxes in CH8 during growth on CDM with amino acids

Metabolite	Flux^*a*^
Uptake	
Lactose	25.1
H_2_O	17.7
Phosphate	0.84
Alanine	0.65
Glutamine	0.39
Proline	0.29
Serine	0.25
Aspartate	0.25
Lysine	0.24
Asparagine	0.18
Leucine	0.17
Glycine	0.15
Guanine	0.13
Uracil	0.13
Valine	0.12
Tryptophan	0.11
Urea	0.08
Isoleucine	0.08
Tyrosine	0.07
Phenylalanine	0.07
Arginine	0.05
Histidine	0.04
Methionine	0.03
Cysteine	0.01
Nicotinate	0.0018
Pantothenate	0.00018
Riboflavin	1.0E−05
Thiamin	1.0E−05
Secretion	
Lactate	40.8
H^+^	39.7
Galactose	24
Glucose	2.5
CO_2_	1.56
Formate	1.09
Acetaldehyde	0.66
Fumarate	0.12
Glycerol	0.024
Glycolaldehyde	1.0E−05

a Flux in mmol/gDW/h from an example parsimonious flux balance analysis (pFBA) simulation.

### Factors affecting the profile of secreted products.

**FIG 4:**
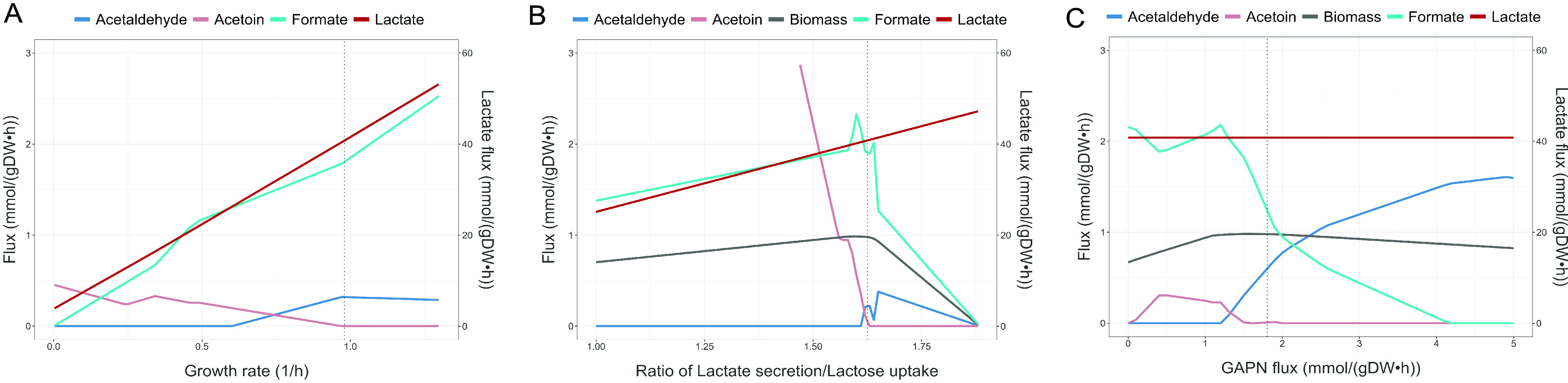
Predicted secretion product profile as a function of three factors, namely, growth rate, ratio of lactate secretion/lactose uptake, and biosynthetic load; biosynthetic load is represented by GAPN (NADP-dependent glyceraldehyde-3-phosphate dehydrogenase) flux. (A) Exchange fluxes as a function of growth rate, with a constant experimentally determined lactate/lactose exchange ratio of 1.625 (as defined by the lactate secretion flux and lactose uptake flux). (B) Exchange fluxes and growth rate as a function of lactate/lactose exchange ratio. Lactose uptake remains constant. (C) Exchange fluxes and growth rate as a function of biosynthetic load with a constant lactate/lactose exchange ratio (1.625). Solid lines indicate pFBA flux predictions. Dotted vertical lines indicate the predicted values with rates determined experimentally (Table 1).

During simulations, a general interdependency was observed for the secretion products lactate, acetoin, formate, acetaldehyde, and ethanol. Several factors were found to influence the secretion profile of these pyruvate-derived metabolites during modeling, such as growth rate, lactate/lactose exchange flux ratio, and biosynthetic load, which was represented as GAPN flux (Fig. 4).

When applying the experimentally measured lactate/lactose flux ratio as a constraint, acetoin and acetaldehyde exchange levels show an inverse relationship. For the growth rate effect, the predefined lactate/lactose flux ratio could differ at higher or lower growth rates; however, the ratio would be expected to be relatively constant within growth rates ranging between 0.8 and 1.0 h^−1^.

Whenever NADH generating and consuming reactions, mainly lactate dehydrogenase (LDH) and glyceraldehyde-3-phosphate dehydrogenase (GAPD), are balanced, excess pyruvate is predicted to be secreted as acetoin. When these reactions are imbalanced, acetaldehyde synthesis can occur at the expense of acetoin. A comparable shift from acetoin to acetaldehyde secretion is observed when the lactate/lactose flux ratio or biosynthetic load increase; lower values result in acetoin secretion, while higher values result in acetaldehyde synthesis. The increased biosynthetic load affects GAPD flux and thereby NADH generation, causing the redox imbalance that triggers acetaldehyde synthesis. Also, formate and acetaldehyde levels display an inverse relationship, as the synthesis of both metabolites generate acetyl-CoA, of which only a finite amount is required. Overall, the effect of changing growth or model parameters on the secretion product profile will ultimately be a combined function of the three factors, and most likely other factors as well, including regulatory. It is important to note that optimal growth is an underlying assumption of the metabolic profiles predicted using parsimonious flux balance analysis (pFBA), while regulatory constraints, which are initially unaccounted for in a stoichiometric model, could lead to different profiles.

### Growth on milk peptides.

Modeling growth in a rich undefined medium, such as milk, is more challenging than the modeling of growth in a defined medium due to uncertainties in medium composition. A key feature of growth in milk is the nature of the main nitrogen source, namely, casein and whey protein, requiring proteolysis for the uptake of derived peptide fragments that can be further cleaved intracellularly to free amino acids. Casein proteins constitute the main protein forms and are the preferred substrate for proteolysis by lactic acid bacteria (32), while cleavage of whey proteins could not be detected during yogurt fermentation (33). Consequently, casein was selected as the substrate during modeling. In the model, the uptake of free amino acids was therefore substituted with the uptake of a theoretical casein-derived peptide, having an amino acid ratio based on the average amino acid composition of casein (34). A measured growth rate of 0.64 h^−1^ for CH8 in milk was used for constraining the solution space, while the lactose/lactate, lactose/galactose, and lactose/glucose exchange flux ratios observed during growth in CDM were retained as constraints (Table 2). To further increase the accuracy of model predictions, experimental data on the concentrations of four fermentation products (Table 3) and most amino acids (see Table S5 in the supplemental material) at the end of CH8 milk fermentation were generated. The secretion of all four fermentation products could be observed at various levels. The experimentally derived fermentation product ratios as well as the ratios between amino acids displaying a net increase in concentration over the course of fermentation (BCAAs, proline, threonine, phenylalanine, and ornithine) were included as model constraints. As proteolysis is the most likely growth limiting factor during dairy fermentation (35), minimization of the casein peptide uptake rate was used as an objective function in model simulations rather than biomass production. Ultimately, the condition-specific model of growth on casein peptide is rather constrained concerning product secretion reactions. Among unconstrained reactions, the secretion of CO_2_, formate, and fumarate are again predicted, as when simulating growth in CDM. For amino acids, some are predicted to be secreted contrary to measurements (glycine, histidine, and leucine). While the observed increase in extracellular amino acids during fermentation could also be a direct effect of extracellular proteolysis, overall, the secretion of certain amino acids and the growth limitation observed in milk indicate that during growth on casein some amino acids are in excess while others could be limiting. Increasing the casein peptide uptake rate in the simulations, and thereby the growth rate, is associated with a corresponding increase in amino acid secretion. Strains displaying higher growth rates could, therefore, theoretically secrete even higher levels of amino acids.

**TABLE 2:** Predicted uptake and secretion fluxes in CH8 with casein peptide as the amino acid source and with a predefined growth rate of 0.64 h^−1^

Metabolite	Flux^*a*^	Range^*b*^
Uptake		
Lactose	22.6	22.6 to 25.1
H_2_O	17.4	17.1 to 21.5
Urea	1.02	0.74 to 1.97
Phosphate	0.544	
Uracil	0.082	0 to 0.082
Caspep	0.0012	
Nicotinate	0.00115	
Pantothenate	0.000115	
Thiamin	6.0E−06	
Riboflavin	6.0E−06	
Secretion		
Lactate	36.8	36.8 to 40.8
H^+^	35.0	35.0 to 41.7
Galactose	21.7	21.7 to 24
Glucose	2.3	2.3 to 2.5
CO_2_	1.47	0.16 to 2.33
Formate	1.03	0.71 to 2.71
Ethanol	0.19	0 to 0.51
Fumarate	0.16	0 to 0.8
Acetoin	0.13	0 to 0.36
Pyruvate	0.07	0 to 0.2
Valine	0.05	0.05 to 0.21
Acetaldehyde	0.04	0 to 0.10
Proline	0.03	0.03 to 0.14
Glycine	0.015	
Isoleucine	0.013	0.013 to 0.06
Leucine	0.009	0.009 to 0.47
Phenylalanine	0.009	0.009 to 0.04
Threonine	0.008	0.008 to 0.04
Ornithine	0.006	0.06 to 0.03
Histidine	0.003	0.003 to 0.38
Cysteine	0.002	0 to 0.002
Tyrosine	2.0E−04	2.0E−04 to 0.27
Glycolaldehyde	6.0E−06	6E−06 to 0.20

a Flux in mmol/gDW/h from an example pFBA simulation.

b Possible flux distribution, as identified by flux variability analysis with 1% variability in casein uptake allowed while maintaining defined growth rate and lactose uptake rate.

**TABLE 3:** Milk fermentation endpoint secretion product concentrations of CH8

Metabolite	Concn (mmol/L)
Acetaldehyde	0.13
Acetoin	0.45
Ethanol	0.63
Pyruvate	0.25

Despite the overall inclusion of multiple secretion constraints, a wide flux range for secretion products can be observed through flux variability analysis (FVA) and also for metabolites that are not secreted in the pFBA solution. This finding might indicate a certain degree of flexibility in metabolism, although most of this flexibility is an effect of the wide feasible range of lactose uptake at this growth rate.

### Amino acid supply and demand during growth in milk.

**FIG 5:**
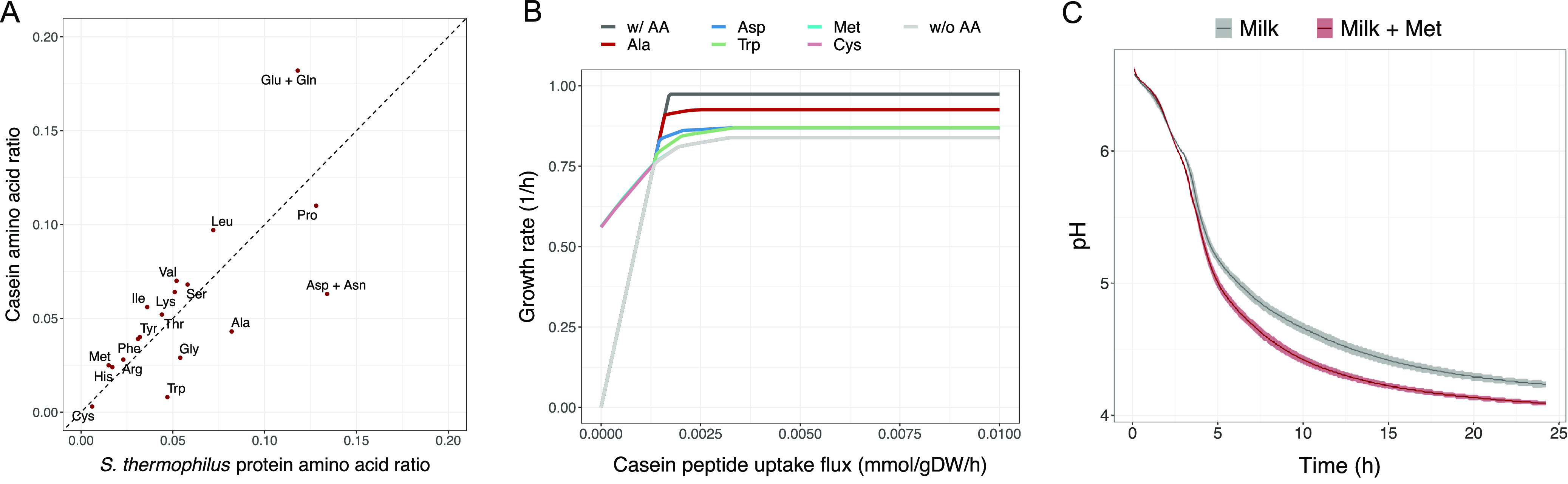
Casein amino acid composition and effect on growth. (A) Comparison of casein amino acid composition ratio and amino acid composition ratio of S. thermophilus total protein content. (B) Effect of casein uptake and free amino acid availability on growth. Graphs represent growth with casein, either without any free amino acids available (w/o AA), with individual free amino acids available (Asp, Met, Ala, Trp, and Cys), or with all amino acids freely available (w/AA) except methionine and cysteine. The interval of casein uptake rate in which sulfur-amino acids are growth limiting is marked in a transparent gray shade. (C) Experimental acidification curves of CH8 milk fermentation, with or without 40 mg/L free methionine supplementation.

An imbalance in the availability of individual amino acids from casein peptide, relative to the biosynthetic demand, can be observed when modeling the growth on casein peptide. A comparison of casein amino acid content (34) with the experimentally determined amino acid composition of S. thermophilus CNRZ1066 cells (5) gives a more direct insight (Fig. 5A). For most amino acids, a good correlation between availability in casein and biosynthetic demand exists; however, for some amino acids, an imbalance is evident. Amino acids present in excess in casein are located generally on the upper (casein) side of the diagonal (Fig. 5A). All these amino acids, except for glutamate, lysine, and serine, are indeed predicted to be secreted by CH8. Regarding the exceptions, glutamate is a frequent substrate for amino acid interconversions through transamination, whereas lysine is further needed for peptidoglycan biosynthesis and a proportion of serine is consumed in the synthesis of tryptophan, cysteine, and glycine. Therefore, these amino acids are also not necessarily expected to be in excess.

The imbalance between casein-derived amino acid supply and S. thermophilus amino acid demand could result in a limitation of growth by specific amino acids. Indeed, at low casein peptide uptake rates, *in silico* growth is limited by sulfur amino acid (cysteine and methionine) availability since to our knowledge they constitute the only significant organic sulfur sources in milk (Fig. 5B). At higher casein peptide uptake rates, growth is instead predicted to be limited by alanine, aspartate, tryptophan, and eventually other amino acids (Fig. 5B). For experimentally validating the hypothesis of sulfur amino acid limitation, CH8 milk fermentations were carried out in the presence or absence of additional methionine. Methionine was chosen as it, unlike cysteine, does not have oxygen scavenging properties. Indeed, the addition of methionine resulted in a marked increase in acidification rate, a proxy for CH8 growth. Overall, the time to pH 4.5, which is an important parameter in dairy fermentations, was reduced from 16 h to 9 h (Fig. 5C).

### Ornithine secretion occurs during milk fermentation.

Transcriptome profiling revealed exceptionally high expression levels of arginine biosynthesis genes during milk fermentation, whereas only minimal arginine biosynthesis was predicted during *in silico* simulation of growth on casein-derived peptide. Besides, based on the amino acid content of both S. thermophilus biomass and casein, an arginine deficiency in milk is not apparent. Thus, the arginine biosynthesis pathway could have an additional function in the supply of other metabolites. Ornithine, for example, is generated as an intermediate in the biosynthesis of arginine. Measurement of extracellular ornithine concentrations during milk fermentation by CH8 revealed an initial decrease followed by a slight increase in the concentration of this amino acid (Table 4). Arginine can also function as a precursor for the synthesis of polyamines, a class of compounds that is ubiquitous among living organisms. One polyamine, putrescine, can be synthesized from arginine in only two reactions. Most S. thermophilus strains harbor a gene annotated as an agmatinase, which is capable of catalyzing the second reaction. To explore this hypothesis, the intracellular and extracellular concentrations of putrescine, together with the extracellular concentrations of ornithine and glutamate (Table 4), were measured for CH8 cultured in milk. None of the polyamines that were measured (putrescine and four other polyamines) could be detected either extracellularly or in crude cell extracts (limit of detection, 0.4 mg/L), rejecting the hypothesis that any excess arginine serves as a precursor for polyamine synthesis in CH8.

**TABLE 4:** Extracellular free amino acid concentrations during milk fermentation

Time (h)	pH	Concn (mg/L) of^*a*^:		
		Arginine (mg/L)	Glutamate (mg/L)	Ornithine (mg/L)
0	6.59	3.4 ± E−3	51.5 ± 0.2	0.6 ± 3E−2
4.5	5.80	N.D.^*b*^	8.9 ± 3.2	N.D.
6.25	5.15	N.D.	2.0 ± 0.1	N.D.
9.5	4.71	N.D.	2.1 ± 0.2	1.1 ± 5E−3

a Average concentration of 3 measurements ± standard deviation.

b N.D., not detected; below detection limit of 0.2 mg/L.

## DISCUSSION

As S. thermophilus is an important workhorse in the dairy industry, and increasingly also in the fermentation of plant-based foods, knowledge about its physiology has both scientific and industrial relevance. Here, a system-wide characterization of S. thermophilus CH8 physiology uncovered novel aspects relating to both carbon and nitrogen metabolism, while examination of both the *in vitro* and *in silico* growth in milk and CDM demonstrated the physiological characteristics of CH8 during fermentation in either medium.

Curation of the previously published S. thermophilus LMG18311 GEM (5) yielded a refined and updated model that provides a foundation for the in-depth understanding of this organism’s metabolism. Considering the limited variation in the distribution of metabolic genes among the studied strains, the reconstructed metabolic network of CH8 can be expected to be broadly representative of the species. A recurring observation in model simulations was the surplus of pyruvate, leading to the secretion of a variety of derived metabolites, including formate, acetaldehyde, acetoin, ethanol, and pyruvate itself, apart from the hallmark lactate secretion. The secretion of these metabolites in milk was either verified experimentally in this work or previously reported by others (4–6, 31, 36). Through modeling, a set of three factors putatively influencing the secretion product profile in S. thermophilus were identified, namely, growth rate, lactate/lactose exchange flux ratio, and biosynthetic load. The main reason underlying the influence of these factors on the fermentation product profile is their effect on NADH, acetyl-CoA, and CO_2_ intracellular pools, as these metabolites take part in the reactions involved in the synthesis of the secretion products. As the three factors reflect general mechanistic constraints during optimal growth, their influence could also be valid easily both under other conditions and in other strains. As for the different distribution of secretion products experimentally observed in milk, it is likely a reflection of regulatory constraints that are not incorporated explicitly in stoichiometric models. Thus, the distribution could also differ in strains with alternative regulatory constraints. While the effects of the three identified factors can be overruled or masked by regulatory mechanisms, the underlying principles of these effects are still at play and should be relevant to consider when, for example, optimizing the synthesis of a certain fermentation product.

One of the secretion products of S. thermophilus, acetaldehyde, is an important flavor compound in yogurt, and yet the exact route of its biosynthesis in this organism has not been resolved definitively. While most organisms synthesize acetaldehyde from acetyl-CoA, through acetaldehyde dehydrogenase activity, this enzyme is absent in S. thermophilus. In this work, constraint-based modeling identified the acetolactate pathway (Fig. 3) as the main source of acetaldehyde formation rather than the threonine aldolase (TA) activity (converting threonine to glycine and acetaldehyde) of the serine hydroxymethyltransferase (SHMT) enzyme as was proposed previously (37). Although various experimental reports on the possible routes of acetaldehyde formation in S. thermophilus exist (4, 37, 38), a clear consensus is absent as it appears that the relative contribution of each pathway varies, possibly with a preference toward the acetolactate pathway.

Nitrogen availability is presumably a growth-limiting factor during growth in milk (35). Modeling amino acid metabolism during growth on the casein peptide provided insights into the growth-limiting effect of amino acids on S. thermophilus. The most pronounced growth limitation relates to the sulfur amino acids cysteine and methionine. The synthesis of both amino acids requires sulfur, which S. thermophilus cannot assimilate from inorganic sources, while the presence of other significant organic sulfur sources in milk is not reported. Thus, unlike other amino acids that can be synthesized *de novo* from glycolytic intermediates, the combined pool of sulfur amino acids largely determines their total availability for protein synthesis. Without exogenous cysteine and methionine, modeling identified that growth is limited by the casein peptide uptake rate, up to a certain level, after which other amino acids, e.g., alanine, aspartate, and tryptophan, become limiting. The predicted sulfur amino acid limitation during growth in milk is in line with the importance of sulfur amino acid synthesis reported previously in S. thermophilus (9). An increase in the expression of sulfur amino acid synthesis genes was also observed, which could indicate the presence of another organic sulfur source or merely reflect cysteine and methionine interconversion. The uptake rate of casein peptides is a function of the proteolytic capabilities of a strain. A key feature in proteolytic S. thermophilus strains is the serine protease PrtS (39), whose presence is subject to significant strain variability (1). This protease is absent in CH8, explaining its lower growth rate in milk than that in CDM. However, strains carrying this protein could display a casein peptide uptake rate that surpasses the growth limitation caused by sulfur amino acids, and all S. thermophilus strains are therefore not necessarily subject to this limitation.

Overall, the current results indicate an imbalance between amino acid supply in the form of peptides and individual amino acid demand. A discrepancy is also observed between pFBA predictions and the expression of genes related to amino acid biosynthesis. Possibly, the average amino acid composition of casein is not an accurate representation of the intracellularly available amino acids. The processes of extracellular proteolysis and peptide transport might lead to a particular intracellular distribution of peptides, thereby also affecting the composition of available intracellular amino acids. Apart from PrtS, certain cell-associated extracellular peptidase activities have been identified (40, 41). An altered amino acid composition could potentially explain contradicting observations obtained from modeling and transcriptome profiling, e.g., within arginine and BCAA biosynthesis pathways.

Alternatively, additional functions for these amino acids, or intermediates in their biosynthesis pathways, that are not described in the reconstructed metabolic network may exist. High arginine biosynthesis gene expression has also been detected previously during S. thermophilus growth in milk using microarray technology (14). Intriguingly, verification of this expression using reverse transcriptase quantitative PCR (RT-qPCR) on *argH* detected a 50-fold upregulation (14), which is similar to the results in our study. Meanwhile, gene expression levels similar to those in the present study have been observed in another RNA-Seq study (18), as well as in an internal study performed on one of our proprietary *prtS+* strains (our unpublished data).

It has been speculated that the mutualistic relationship between S. thermophilus and *L. delbrueckii* subsp. *bulgaricus* during milk fermentation includes the exchange of ornithine produced by S. thermophilus and subsequent ornithine-derived putrescine produced by *L. delbrueckii* subsp. *bulgaricus* (42). The increase in extracellular ornithine concentration over time observed in this study indicates that the ornithine/putrescine exchange hypothesis could be correct, although the final ornithine concentration is low. Possibly, the ornithine production of S. thermophilus would be enhanced during coculture. Increased arginine biosynthesis gene expression was indeed observed during coculture with *L. delbrueckii* subsp. *bulgaricus* (15).

Modeling the mutualistic relationship between *L. delbrueckii* subsp. *bulgaricus* and S. thermophilus would be a natural continuation of this study and has relevance both from an industrial and an evolutionary perspective. Several interactions have already been described (14, 15, 25), of which some have been evidenced and some remain as hypotheses. Future coculture modeling can help substantiate these aspects, quantify the interactions, and perhaps identify novel relationships.

The growth of S. thermophilus in both CDM and milk was examined here through a combined systems biology approach. This examination led to a better understanding of the characteristics of growth in its main niche, milk, at the level of both carbon and nitrogen metabolism, where principles governing the secretion product profile but also nitrogen limitation were uncovered. The availability of a refined and updated GEM capable of accurately simulating growth and metabolite secretion by S. thermophilus enables the future system-level mechanistic understanding of S. thermophilus physiology. Considering the disparity in amino acid supply and demand during growth in milk, studies on the effect of proteolysis and peptide uptake could provide more detailed knowledge on the actual distribution of intracellularly available amino acids. The knowledge presented here on the proposed mechanisms governing the secretion product profile could as well be harnessed to fine-tune or engineer strains displaying a desirable product profile. Moreover, the new GEM could provide the foundation for the characterization of the mutualistic relationship between S. thermophilus and *L. delbrueckii* subsp. *bulgaricus* in greater detail.

## MATERIALS AND METHODS

### Strains, media, and culture conditions.

Streptococcus thermophilus CH8 is a proprietary strain, which was obtained from the Chr. Hansen Culture Collection. Strains were cultured in media consisting of either a chemically defined medium (CDM) or milk. A CDM for S. thermophilus has been described previously (43) and was modified in this study. The medium contained (per liter of demineralized water) 20 g lactose; 1 g Na-acetate; 0.6 g NH_4_-citrate; 3 g KH_2_PO_4_; 2.5 g K_2_HPO_4_; 0.24 g urea; 0.42 g NaHCO_3_; 0.2 g MgCl_2_·6 H_2_O; 0.05 g CaCl_2_·2 H_2_O; 0.028 g MnSO_4_·H_2_O; 0.005 g FeCl_2_·4H_2_O; 1-mL trace element solution; 10-mL vitamin solution; 0.01 g of each of the four nucleobases adenine, guanine, uracil, and xanthine; 0.5 g cysteine; and 0.04 g of each of the l-amino acids alanine, arginine, asparagine, aspartic acid, glutamine, glutamic acid, glycine, histidine, isoleucine, leucine, lysine, methionine, phenylalanine, proline, serine, threonine, tryptophan, tyrosine, and valine. The trace element solution consisted of (per liter of demineralized water) 77 mM HCl, 1.5 g FeCl_2_ × 4 H_2_O, 70 mg ZnCl_2_, 100 mg MnCl_2_·4H_2_O, 6 mg H_3_BO_3_, 190 mg CoCl_2_·6 H_2_O, 2 mg CuCl_2_·2 H_2_O, and 24 mg NiCl_2_·6 H_2_O while the vitamin solution consisted of (per liter of demineralized water) 100 mg pyridoxine-HCl, 50 mg p-aminobenzoic acid, 50 mg nicotinic acid, 400 mg Ca-DL-panthothenate, 50 mg thiamine, 50 mg lipoic acid, 50 mg riboflavin, 20 mg biotin, 20 mg folic acid, and 1 mg vitamin B_12_. The medium was adjusted to pH 6.6 and distributed as 50-mL aliquots in 250-mL crimp-top serum bottles with subsequent flushing of the headspace with N_2_ gas for 10 min. The medium was sterilized by autoclaving at 121°C for 20 min. Prior to inoculation, the medium was supplemented with sterile, anaerobic solutions of lactose, MgCl_2_·6 H_2_O, CaCl_2_·2 H_2_O, urea, trace elements, vitamins, amino acids, and nucleobases. As the final step, a cysteine-HCl solution was added to lower the redox potential. For growth in milk, skim milk powder (Arla Foods amba, Denmark), containing around 60% protein and 1% fat, was reconstituted at a dry matter level of 9.5% in distilled water and pasteurized at 99°C for 30 min, followed by cooling. Following the aseptic transfer of milk to a sterile crimp-top serum bottle, dithiothreitol was added as a reducing agent to a final concentration of 0.15 g/L, and the bottle headspace was flushed with N_2_ for 20 min. All fermentations were conducted under anoxic conditions. For inoculum preparation, bacteria were streaked from glycerol stock onto M17 (44) agar plates and incubated anaerobically overnight at 37°C, where after a single colony was inoculated into the CDM and incubated anaerobically overnight at 37°C. From this preparation, an inoculum was generated by inoculating the CDM at the desired level to obtain exponentially growing cells following overnight incubation at 37°C. Growth was monitored by following the optical density at 600 nm (OD_600_) of the cultures.

### Genome sequencing and analysis.

Genome sequencing was performed using both Illumina short-read and Oxford Nanopore Technologies (ONT;, Oxford, UK) long-read sequencing technologies. For short-read sequencing, genomic DNA was extracted using the DNeasy blood and tissue kit (Qiagen, Hilden, Germany). The DNA was then fragmented using a BioRuptor instrument (Diagenode Inc., Denville, NJ) aiming at an insert size of 800 bp. A sequencing library was prepared using the Kapa HTP kit (Roche, Basel, Switzerland), according to the manufacturer’s protocol. Sequencing was done using MiSeq reagent kit v2 on a MiSeq instrument (Illumina, San Diego, CA), with a read length of 2 × 250 bp. For long-read sequencing, genomic DNA was purified using phenol-chloroform extraction followed by ethanol precipitation (45). Prior to library preparation, DNA was subjected to an additional purification step with AMPure XP beads (Beckman Coulter, Brea, CA), following the manufacturer’s protocol. The sequencing library was prepared using the 1D genomic DNA by ligation kit (SQK-LSK108; ONT), following the workflow suggested by the manufacturer. The prepared library was sequenced on a MinION instrument (ONT).

A hybrid genome assembly approach based on both Illumina and ONT reads was followed using SPAdes 3.8.0 (46), with a coverage of 45-fold for ONT reads and 115-fold for Illumina reads. Low coverage contigs (<1.5 coverage) were removed and a long-read-only assembly was created, using Canu v. 1.3 (47) to resolve one final gap.

Open reading frames (ORFs) in the assembled genome were identified and annotated functionally using the RAST pipeline (48). The phylogeny of S. thermophilus strains was reconstructed by first aligning 534 core genes, identified using Roary (49) with PRANK (50) alignment. Poorly aligned positions were then eliminated with Gblocks (51), and maximum-likelihood phylogeny was inferred using RAxML-NG, which is RaxML based (52), with the GTRGAMMA model, 50 random starting trees, and 100 bootstrap replicates. Phylogenetic trees were visualized using FigTree (53), and strain NCTC8618 of *S. salivarius*, a closely related species, was included as an outgroup. For a pangenome analysis, protein ortholog detection was performed using proteinortho (54) employing a sequence identity of 95%, coverage of 90%, and similarity cutoff of 1. To avoid annotation bias, the ortholog comparison was based on a PGAP (55) annotated version of the CH8 genome and the refseq version of the public genomes (refseq genomes are annotated with PGAP). Subsequently, proteins specific to CH8, or rare among other strains, and orthologs absent in CH8 while present in the majority (18) of strains were identified, and differences relating to metabolic genes were highlighted. Hierarchical clustering based on ortholog distribution was performed in R using the Jaccard distance and unweighted pair group method with arithmetic mean (UPGMA) linkage method.

### Isolation and processing of RNA.

For growth in both CDM and milk, a preculture grown anaerobically overnight at 37°C in CDM was used to inoculate the desired medium to a final OD_600_ of 0.05, and the resulting cultures were incubated at 37°C. Cultivations were performed in triplicates in each medium, and cells were harvested at two time points, as follows: at an OD_600_ of ca. 0.6 to 0.7 and 1.0 to 1.2 for the cultures in CDM and around pH 5.92 and 5.37 for the cultures in milk. A 1:2 volume of cell culture to RNAprotect bacterial reagent (Qiagen) was mixed rigorously at cell harvest for both CDM and milk samples. For CDM growth, cell pellets were harvested by centrifugation. To harvest cells grown in milk, samples were subjected to mild sonication for 30 s, and the procedure for cell-milk separation was adapted from previous reports (56, 57). To a 12-mL milk:RNAprotect mix, 4 mL 1 M Na-citrate (pH 7.0) and a 1.56-mL saline solution (0.145 NaCl, 0.016 M Na-β-glycerophospate, and 0.1% Tween 80 [pH 7.0]) were added, mixed, incubated at room temperature (RT) for 5 min, and centrifuged at 10,000 × *g* for 5 min at 4°C. The cell pellet was washed with 1 mL extraction buffer (5 mM NaPO_4_ and 1 mM EDTA [pH 7]) and stored subsequently at −80°C. The cell pellet was dissolved in Tris-EDTA (TE) buffer containing lysozyme (15 mg/mL), proteinase K (20 mg/mL), and mutanolysin (250U/μL) and shaken at 37°C and 1,400 rpm for 10 min. The subsequent RNA extraction procedure was performed with an RNeasy minikit (Qiagen) as per the manufacturer’s instructions, including the removal of DNA with DNase I. The quality of the total RNA was evaluated using a bioanalyzer (Agilent), before using the Ribo-Zero rRNA removal kit for bacteria (Illumina) for rRNA depletion, according to the manufacturer’s protocol. Sequencing thereafter was performed at Center for Biosustainability (Technical University of Denmark, Denmark), where a TruSeq RNA library prep kit (2 × 75 bp; Illumina) was used for library preparation and paired-end sequencing was performed on an Illumina NextSeq instrument.

### RNA-Seq data analysis.

Detailed sequencing statistics can be found in Table S2 in the supplemental material. The obtained raw reads were trimmed with Trimmomatic (58) using default parameters, with the resulting unpaired reads removed. All remaining reads were mapped to an initial draft version of the CH8 genome, obtained through a hybrid genome assembly approach. Read mapping was performed with CLC Genomics Workbench (Qiagen), with the default parameters, and unique gene counts were extracted. A differential gene expression analysis was performed using DESeq2 (59) within the SARTools framework (60) with default parameters. Values were mapped to genes annotated on the complete genome, by the principle of orthology. Transcriptome data were visualized on CH8 metabolic maps with Escher software (61).

### Genome-scale metabolic modeling.

The GEM of S. thermophilus CH8 iRZ476 was created based on a comparative genomics approach using the GEM reconstruction tool Metadraft v. 0.8.1 (62). Briefly, protein orthologs were identified for CH8 and five selected reference strains with publicly available GEMs. Reactions associated with orthologs in the reference strains were included in the CH8 model after being subjected to manual filtering, thus excluding reactions implausible in S. thermophilus. Reference strain species included S. thermophilus LMG18311, Lactobacillus plantarum, Lactococcus lactis, Bacillus subtilis, Staphylococcus aureus, and Escherichia coli (5, 26–30). Compared with the LMG18311 model, 22, 51, and 27 reactions were removed, added, or constrained, respectively. The GEM is provided in File S1 in the supplemental material. Medium formulations included a medium similar to the defined CDM, with either amino acids or casein-derived peptides as the nitrogen source. For growth on casein peptides, an average peptide, designated casein peptide, was formulated with an average amino acid composition based on previous determinations of casein composition (34). Optimal growth states were identified using pFBA and flux variability analysis (FVA) within the COBRApy framework (63). Uptake and secretion rates of lactose, glucose, galactose, and lactate were determined for growth in CDM by time course metabolite measurement and metabolite yield calculation through linear regression based on the equation q_p_ = Y_p/x_ · μ, where q_p_ is the rate of product formation, Y_p/x_ the specific product yield coefficient, and μ the growth rate. Rates were calculated at intervals in which a linear relationship between growth rate and metabolite concentration was observed. For growth in CDM, growth rate was maximized during modeling, while for growth on the casein peptide, growth rate was constrained to 0.64 according to experimental data, and instead casein peptide uptake was minimized. Additionally, for modeling growth on casein peptide, experimentally observed ratios between secreted metabolites of acetaldehyde, acetoin, ethanol, and pyruvate, as well as experimentally observed ratios between accumulating amino acids, proline, ornithine, threonine, isoleucine, valine, and phenylalanine, were applied as constraints.

### Fermentation kinetics in CDM and milk.

For simulating growth and metabolite formation in CDM and milk, detailed fermentation kinetics were obtained by growing CH8 principally as described above. For CDM fermentation, anaerobic growth was performed as described, in duplicates, although the medium contained twice the amino acid concentrations, apart from cysteine that remained at 0.5 g/L. Samples for metabolite analysis were collected and mixed 5:1 with 2 M H_2_SO_4_. Milk fermentation was here performed in 200-mL screw-cap bottles fitted with pH sensors. Milk acidification was followed by monitoring the changes in pH for up to 30 h at 40°C, using an iCinac system (AMS Alliance, Frepillon, France). Growth was followed by monitoring the OD_600_ of samples clarified with borate-EDTA buffer (pH 8.0). For OD_600_ of <0.2 measurements, 0.5 mL of a milk sample was treated with 2 M borate–200 mM EDTA (pH 8.0) as described by Christensen and Steele (64). From that point onward, 0.1 mL of the sample was mixed with 0.9 mL of 0.5 M borate–10 mM EDTA (pH 8.0), and the OD_600_ of the mixture was determined after a 30-min incubation at room temperature. Absolute OD_600_ values of the cultures were corrected by deducting the OD_600_ of noninoculated B-milk clarified in the same way. For methionine supplementation experiments, fermentations were performed with or without methionine added at a concentration of 80 mg/L.

### Metabolite analysis.

Extracellular levels of acetaldehyde, acetoin, ethanol, and pyruvate in milk samples from iCinac fermentations were analyzed in-house by gas chromatography coupled to flame ionization detection as described previously (65), with modifications. For acetaldehyde, acetoin, and ethanol analysis, a 1-mL sample was added to a 20-mL headspace vial already containing 200 μL 2 M sulfuric acid in Milli-Q water and the vial capped immediately. For pyruvic acid analysis, a 600-μL sample was added to a 20-mL headspace vial already containing 400 μL 300 mg d-α-hydroxyisovaleric acid/L in saturated NaHSO_4_. Subsequently, 0.5 mL ice-cold methanol was added and the vial capped immediately. For quantification, a standard addition with sodium pyruvate was done to a pool of samples using d-α-hydroxyisovaleric acid as the internal standard. The gas chromatograph (Autosystem XL; Perkin-Elmer) was equipped with a flame ionization detector and a free fatty acid phase (FFAP) column (25 m × 0.2 mm × 0.3 μm; 19091F-102; Agilent Technologies). The gas chromatograph was connected to a headspace sampler (TurboMatrix 110 Trap; Perkin Elmer). The operating parameters of the gas chromatograph were as follows: 32 lb/in^2^ of head pressure, 55 mL/min helium flow rate, 180°C injector temperature, and 200°C detector temperature. The oven temperature was held at 60°C for 2 min; the temperature increased by 45°C/min up to 230°C. The parameters of the headspace sampler were as follows (values in parentheses were used for samples prepared according to the procedure for pyruvic acid determination): 70°C incubation temperature for 36.5 min (77 min), 180°C needle temperature, 210°C transfer line temperature, 1 min of pressurization time, and 0.02 min of injection time with split ratio 4.5. Unless stated otherwise, chemicals used were of analytical grade.

To track arginine-related metabolites, free amino acid concentrations were measured for both CH8 cell extracts and supernatants that were obtained during milk fermentations. Precultures and cultures were prepared as described under “Isolation and processing of RNA.” For the extracellular measurement of free amino acids, samples were collected and frozen until analyzed. For the preparation of cell extracts, 50 mL milk fermentate was subjected to cold washing for the separation of cells from the milk protein matrix., using previously described wash buffers (57), with modifications. Initially, samples were washed with 25 mL 1 M sodium citrate and 10-mL saline solution. Two subsequent washes were performed with a mixture of 25-mL extraction solution and 25-mL borate solution (0.5 M borate and 10 mM EDTA). The resulting cell pellet was resuspended in 1 mL 0.05 M Na-phosphate buffer (pH 7), and cells were lysed by sonication (Misonix 3000). Concentrations of free arginine, ornithine, and glutamate in extracellular samples and cell extract samples were analyzed by Ansynth Service B.V. (The Netherlands) using classical ion-exchange liquid chromatography with postcolumn ninhydrin derivatization and photometric detection. Polyamine and free amino acid levels were analyzed in-house by gas chromatography coupled to mass spectrometry with electronic ionization. Derivatization was performed through alkylation with methylchloroformate (66).

### Statistics and reproducibility.

Differential expression analysis was performed with the DESeq2 R package applying the default median normalization and the parametric fit type, and a Benjamini-Hochberg correction was performed. A principal-component analysis of samples was performed on transformed (variance stabilizing transformation) count values. Differential gene expression was considered significant if the *q* value was below 0.05 and log_2_ fold change was above |±1|. Unless stated otherwise, all examples of differential gene expression mentioned adhere to these criteria.

### Data availability.

The annotated genome sequence was deposited at NCBI GenBank (CP089060), while the RNA-Seq reads were submitted to the Sequence Read Archive (SRR15427001 to SRR15427012).

## References

[1] Hols P, Hancy F, Fontaine L, Grossiord B, Prozzi D, Leblond-Bourget N, Decaris B, Bolotin A, Delorme C, Dusko Ehrlich S, Guédon E, Monnet V, Renault P, Kleerebezem M. 2005. New insights in the molecular biology and physiology of *Streptococcus thermophilus* revealed by comparative genomics. FEMS Microbiol Rev 29:435–463. 10.1016/j.femsre.2005.04.008. 16125007

[2] Orla-Jensen S. 1919. The lactic acid bacteria. A. F. Høst og Søn, Copenhagen, Denmark.

[3] Bolotin A, Quinquis B, Renault P, Sorokin A, Ehrlich SD, Kulakauskas S, Lapidus A, Goltsman E, Mazur M, Pusch GD, Fonstein M, Overbeek R, Kyprides N, Purnelle B, Prozzi D, Ngui K, Masuy D, Hancy F, Burteau S, Boutry M, Delcour J, Goffeau A, Hols P. 2004. Complete sequence and comparative genome analysis of the dairy bacterium *Streptococcus thermophilus*. Nat Biotechnol 22:1554–1558. 10.1038/nbt1034. 15543133 PMC7416660

[4] Andreas O, Jacques-Edouard Germond A, Chaintreau A. 2000. Origin of acetaldehyde during milk fermentation using 13C-labeled precursors. J Agric Food Chem 48:1512–1517. 10.1021/jf9904867. 10820052

[5] Pastink MI, Teusink B, Hols P, Visser S, de Vos WM, Hugenholtz J. 2009. Genome-scale model of Streptococcus thermophilus LMG18311 for metabolic comparison of lactic acid bacteria. Appl Environ Microbiol 75:3627–3633. 10.1128/AEM.00138-09. 19346354 PMC2687286

[6] Teraguchi S, Ono J, Kiyosawa I, Okonogi S. 1987. Oxygen uptake activity and aerobic metabolism of Streptococcus thermophilus STH450. J Dairy Sci 70:514–523. 10.3168/jds.S0022-0302(87)80036-X. 3584599

[7] Courtin P, Monnet V, Rul F. 2002. Cell-wall proteinases PrtS and PrtB have a different role in *Streptococcus thermophilus*/*Lactobacillus bulgaricus* mixed cultures in milk. Microbiology (Reading) 148:3413–3421. 10.1099/00221287-148-11-3413. 12427933

[8] Delorme C, Bartholini C, Bolotine A, Ehrlich SD, Renault P. 2010. Emergence of a cell wall protease in the Streptococcus thermophilus population. Appl Environ Microbiol 76:451–460. 10.1128/AEM.01018-09. 19915034 PMC2805209

[9] Herve-Jimenez L, Guillouard I, Guedon E, Gautier C, Boudebbouze S, Hols P, Monnet V, Rul F, Maguin E. 2008. Physiology of *Streptococcus thermophilus* during the late stage of milk fermentation with special regard to sulfur amino-acid metabolism. Proteomics 8:4273–4286. 10.1002/pmic.200700489. 18814336

[10] Garault P, Letort C, Juillard V, Monnet V. 2000. Branched-chain amino acid biosynthesis is essential for optimal growth of *Streptococcus thermophilus* in milk. Appl Environ Microbiol 66:5128–5133. 10.1128/AEM.66.12.5128-5133.2000. 11097879 PMC92433

[11] Szymczak P, Janzen T, Neves AR, Kot W, Hansen LH, Lametsch R, Neve H, Franz CMAP, Vogensen FK. 2017. Novel variants of Streptococcus thermophilus bacteriophages are indicative of genetic recombination among phages from different bacterial species. Appl Environ Microbiol 83:e02748-16. 10.1128/AEM.02748-16. 28039135 PMC5311409

[12] Philippe C, Levesque S, Dion MB, Tremblay DM, Horvath P, Lüth N, Cambillau C, Franz C, Neve H, Fremaux C, Heller KJ, Moineau S. 2020. Novel genus of phages infecting Streptococcus thermophilus: genomic and morphological characterization. Appl Environ Microbiol 86:e00227-20. 10.1128/AEM.00227-20. 32303549 PMC7301855

[13] Mahony J, van Sinderen D. 2014. Current taxonomy of phages infecting lactic acid bacteria. Front Microbiol 5:7. 10.3389/fmicb.2014.00007. 24478767 PMC3900856

[14] Herve-Jimenez L, Guillouard I, Guedon E, Boudebbouze S, Hols P, Monnet V, Maguin E, Rul F. 2009. Postgenomic analysis of *Streptococcus thermophilus* cocultivated in milk with *Lactobacillus delbrueckii* subsp. *bulgaricus*: involvement of nitrogen, purine, and iron metabolism. Appl Environ Microbiol 75:2062–2073. 10.1128/AEM.01984-08. 19114510 PMC2663229

[15] Sieuwerts S, Molenaar D, Van Hijum SAFT, Beerthuyzen M, Stevens MJA, Janssen PWM, Ingham CJ, De Bok FAM, De Vos WM, Van Hylckama Vlieg JET. 2010. Mixed-Culture transcriptome analysis reveals the molecular basis of mixed-culture growth in *Streptococcus thermophilus* and *Lactobacillus bulgaricus*. Appl Environ Microbiol 76:7775–7784. 10.1128/AEM.01122-10. 20889781 PMC2988612

[16] Goh YJ, Goin C, O'Flaherty S, Altermann E, Hutkins R. 2011. Specialized adaptation of a lactic acid bacterium to the milk environment: the comparative genomics of *Streptococcus thermophilus* LMD-9. Microb Cell Fact 10:S22. 10.1186/1475-2859-10-S1-S22. 21995282 PMC3231929

[17] Li J, Bi Y, Dong C, Yang J, Liang W. 2011. Transcriptome analysis of adaptive heat shock response of *Streptococcus thermophilus*. PLoS One 6:e25777. 10.1371/journal.pone.0025777. 22022447 PMC3192767

[18] Wu Q, Shah NP. 2018. Comparative mRNA-Seq analysis reveals the improved EPS production machinery in *Streptococcus thermophilus* ASCC 1275 during optimized milk fermentation. Front Microbiol 9:445. 10.3389/fmicb.2018.00445. 29593689 PMC5859087

[19] Padmanabhan A, Tong Y, Wu Q, Zhang J, Shah NP. 2018. Transcriptomic insights into the growth phase- and sugar-associated changes in the exopolysaccharide production of a high EPS-producing *Streptococcus thermophilus* ASCC 1275. Front Microbiol 9:1919. 10.3389/fmicb.2018.01919. 30177921 PMC6109772

[20] Qiao Y, Leng C, Liu G, Zhang Y, Lv X, Chen H, Sun J, Feng Z. 2019. Transcriptomic and proteomic profiling revealed global changes in *Streptococcus thermophilus* during pH-controlled batch fermentations. J Microbiol 57:769–780. 10.1007/s12275-019-8604-y. 31201725

[21] Wu Q, Chu H, Padmanabhan A, Shah NP. 2019. Functional genomic analyses of exopolysaccharide-producing *Streptococcus thermophilus* ASCC 1275 in response to milk fermentation conditions. Front Microbiol 10:1975. 10.3389/fmicb.2019.01975. 31507577 PMC6716118

[22] Proust L, Haudebourg E, Sourabié A, Pedersen M, Besançon I, Monnet V, Juillard V. 2020. Multi-omics approach reveals how yeast extract peptides shape Streptococcus thermophilus metabolism. Appl Environ Microbiol 86:e01446-20. 10.1128/AEM.01446-20. 32769193 PMC7642077

[23] Rau MH, Zeidan AA. 2018. Constraint-based modeling in microbial food biotechnology. Biochem Soc Trans 46:249–260. 10.1042/BST20170268. 29588387 PMC5906707

[24] WHO, FAO. 2010. Codex standard for fermented milks (CODEX STAN 243-2003). Food and Agriculture Organization of the United Nations and World Health Organization, Rome, Italy.

[25] Crittenden RG, Martinez NR, Playne MJ. 2003. Synthesis and utilisation of folate by yoghurt starter cultures and probiotic bacteria. Int J Food Microbiol 80:217–222. 10.1016/s0168-1605(02)00170-8. 12423923

[26] Teusink B, Wiersma A, Molenaar D, Francke C, de Vos WM, Siezen RJ, Smid EJ. 2006. Analysis of growth of *Lactobacillus plantarum* WCFS1 on a complex medium using a genome-scale metabolic model. J Biol Chem 281:40041–40048. 10.1074/jbc.M606263200. 17062565

[27] Flahaut NAL, Wiersma A, van de Bunt B, Martens DE, Schaap PJ, Sijtsma L, Dos Santos VAM, de Vos WM. 2013. Genome-scale metabolic model for *Lactococcus lactis* MG1363 and its application to the analysis of flavor formation. Appl Microbiol Biotechnol 97:8729–8739. 10.1007/s00253-013-5140-2. 23974365

[28] Orth JD, Conrad TM, Na J, Lerman JA, Nam H, Feist AM, Palsson BØ. 2011. A comprehensive genome-scale reconstruction of *Escherichia coli* metabolism. Mol Syst Biol 7:535. 10.1038/msb.2011.65. 21988831 PMC3261703

[29] Becker SA, Palsson BØ. 2005. Genome-scale reconstruction of the metabolic network in Staphylococcus aureus N315: an initial draft to the two-dimensional annotation. BMC Microbiol 5:8. 10.1186/1471-2180-5-8. 15752426 PMC1079855

[30] Henry CS, Zinner JF, Cohoon MP, Stevens RL. 2009. iBsu1103: a new genome-scale metabolic model of *Bacillus subtilis* based on SEED annotations. Genome Biol 10:R69. 10.1186/gb-2009-10-6-r69. 19555510 PMC2718503

[31] Perez PF, de Antoni GL, Añon MC. 1991. Formate production by *Streptococcus thermophilus* cultures. J Dairy Sci 74:2850–2854. 10.3168/jds.S0022-0302(91)78465-8.

[32] Rodríguez-Serrano GM, García-Garibay M, Cruz-Guerrero AE, Gómez-Ruiz L, Ayala-Niño A, Castañeda-Ovando A, González-Olivares LG. 2018. Proteolytic system of Streptococcus thermophilus. J Microbiol Biotechnol 28:1581–1588. 10.4014/jmb.1807.07017. 30196594

[33] Bertrand-Harb C, Ivanova IV, Dalgalarrondo M, Haertllé T. 2003. Evolution of β-lactoglobulin and α-lactalbumin content during yoghurt fermentation. Int Dairy J 13:39–45. 10.1016/S0958-6946(02)00140-1.

[34] Lauer BH, Baker BE. 1977. Amino acid composition of casein isolated from the milks of different species. Can J Zool 55:231–236. 10.1139/z77-026. 13923

[35] Savijoki K, Ingmer H, Varmanen P. 2006. Proteolytic systems of lactic acid bacteria. Appl Microbiol Biotechnol 71:394–406. 10.1007/s00253-006-0427-1. 16628446

[36] Akyol I, Ozcelik FG, Karakas-Sen A, Ozkose E, Gezginc Y, Ekinci MS. 2015. Cloning and overexpression of the als, pflA, and adhB genes in *Streptococcus thermophilus* and their effects on metabolite formation. Mol Biotechnol 57:923–930. 10.1007/s12033-015-9882-1. 26280324

[37] Chaves ACSD, Fernandez M, Lerayer ALS, Mierau I, Kleerebezem M, Hugenholtz J. 2002. Metabolic engineering of acetaldehyde production by *Streptococcus thermophilus*. Appl Environ Microbiol 68:5656–5662. 10.1128/AEM.68.11.5656-5662.2002. 12406762 PMC129919

[38] Wilkins DW, Schmidt RH, Shireman RB, Smith KL, Jezeski JJ. 1986. Evaluating acetaldehyde synthesis from L-[14C(U)] threonine by *Streptococcus thermophilus* and *Lactobacillus bulgaricus*. J Dairy Sci 69:1219–1224. 10.3168/jds.S0022-0302(86)80526-4.

[39] Shahbal S, Hemme D, Desmazeaud M. 1991. High cell wall-associated proteinase activity of some *Streptococcus thermophilus* strains (H-strains) correlated with a high acidification rate in milk. Lait 71:351–357. 10.1051/lait:1991327.

[40] Hafeez Z, Cakir-Kiefer C, Girardet J-M, Lecomte X, Paris C, Galia W, Dary A, Miclo L. 2015. New insights into the proteolytic system of *Streptococcus thermophilus*: use of isracidin to characterize cell-associated extracellular peptidase activities. J Agric Food Chem 63:7522–7531. 10.1021/acs.jafc.5b01647. 26193375

[41] Boulay M, Metton C, Mézange C, Oliveira Correia L, Meylheuc T, Monnet V, Gardan R, Juillard V. 2021. Three distinct proteases are responsible for overall cell surface proteolysis in Streptococcus thermophilus. Appl Environ Microbiol 87:e0129221. 10.1128/AEM.01292-21. 34550764 PMC8580001

[42] van de Guchte M, Penaud S, Grimaldi C, Barbe V, Bryson K, Nicolas P, Robert C, Oztas S, Mangenot S, Couloux A, Loux V, Dervyn R, Bossy R, Bolotin A, Batto J-M, Walunas T, Gibrat J-F, Bessières P, Weissenbach J, Ehrlich SD, Maguin E. 2006. The complete genome sequence of *Lactobacillus bulgaricus* reveals extensive and ongoing reductive evolution. Proc Natl Acad Sci USA 103:9274–9279. 10.1073/pnas.0603024103. 16754859 PMC1482600

[43] Letort C, Juillard V. 2001. Development of a minimal chemically-defined medium for the exponential growth of Streptococcus thermophilus. J Appl Microbiol 91:1023–1029. 10.1046/j.1365-2672.2001.01469.x. 11851809

[44] Terzaghi BE, Sandine WE. 1975. Improved medium for lactic streptococci and their bacteriophages. Appl Microbiol 29:807–813. 10.1128/am.29.6.807-813.1975. 16350018 PMC187084

[45] Sambrook J, Russell DW. 2006. Purification of nucleic acids by extraction with phenol:chloroform. Cold Spring Harb Protoc 2006:pdb.prot4455. 10.1101/pdb.prot4455. 22485786

[46] Bankevich A, Nurk S, Antipov D, Gurevich AA, Dvorkin M, Kulikov AS, Lesin VM, Nikolenko SI, Pham S, Prjibelski AD, Pyshkin AV, Sirotkin AV, Vyahhi N, Tesler G, Alekseyev MA, Pevzner PA. 2012. SPAdes: a new genome assembly algorithm and its applications to single-cell sequencing. J Comput Biol 19:455–477. 10.1089/cmb.2012.0021. 22506599 PMC3342519

[47] Koren S, Walenz BP, Berlin K, Miller JR, Bergman NH, Phillippy AM. 2017. Canu: scalable and accurate long-read assembly via adaptive k-mer weighting and repeat separation. Genome Res 27:722–736. 10.1101/gr.215087.116. 28298431 PMC5411767

[48] Overbeek R, Olson R, Pusch GD, Olsen GJ, Davis JJ, Disz T, Edwards RA, Gerdes S, Parrello B, Shukla M, Vonstein V, Wattam AR, Xia F, Stevens R. 2014. The SEED and the Rapid Annotation of microbial genomes using Subsystems Technology (RAST). Nucleic Acids Res 42:D206–D214. 10.1093/nar/gkt1226. 24293654 PMC3965101

[49] Page AJ, Cummins CA, Hunt M, Wong VK, Reuter S, Holden MTG, Fookes M, Falush D, Keane JA, Parkhill J. 2015. Roary: rapid large-scale prokaryote pan genome analysis. Bioinformatics 31:3691–3693. 10.1093/bioinformatics/btv421. 26198102 PMC4817141

[50] Löytynoja A, Goldman N. 2005. An algorithm for progressive multiple alignment of sequences with insertions. Proc Natl Acad Sci USA 102:10557–10562. 10.1073/pnas.0409137102. 16000407 PMC1180752

[51] Talavera G, Castresana J, Kjer K, Page R, Sullivan J. 2007. Improvement of phylogenies after removing divergent and ambiguously aligned blocks from protein sequence alignments. Syst Biol 56:564–577. 10.1080/10635150701472164. 17654362

[52] Stamatakis A. 2014. RAxML version 8: a tool for phylogenetic analysis and post-analysis of large phylogenies. Bioinformatics 30:1312–1313. 10.1093/bioinformatics/btu033. 24451623 PMC3998144

[53] Rambaut A. 2012. FigTree v.1.4.3. University of Oxford, Oxford, UK.

[54] Lechner M, Findeiss S, Steiner L, Marz M, Stadler PF, Prohaska SJ. 2011. Proteinortho: detection of (Co-)orthologs in large-scale analysis. BMC Bioinformatics 12:124. 10.1186/1471-2105-12-124. 21526987 PMC3114741

[55] Li W, O'Neill KR, Haft DH, DiCuccio M, Chetvernin V, Badretdin A, Coulouris G, Chitsaz F, Derbyshire MK, Durkin AS, Gonzales NR, Gwadz M, Lanczycki CJ, Song JS, Thanki N, Wang J, Yamashita RA, Yang M, Zheng C, Marchler-Bauer A, Thibaud-Nissen F. 2021. RefSeq: expanding the Prokaryotic Genome Annotation Pipeline reach with protein family model curation. Nucleic Acids Res 49:D1020–D1028. 10.1093/nar/gkaa1105. 33270901 PMC7779008

[56] Guimont C, Chopard M-A, Gaillard J-L, Chamba J-F. 2002. Comparative study of the protein composition of three strains of *Streptococcus thermophilus* grown either in M17 medium or in milk. Lait 82:645–656. 10.1051/lait:2002039.

[57] Derzelle S, Bolotin A, Mistou M-Y, Rul F. 2005. Proteome analysis of *Streptococcus thermophilus* grown in milk reveals pyruvate formate-lyase as the major upregulated protein. Appl Environ Microbiol 71:8597–8605. 10.1128/AEM.71.12.8597-8605.2005. 16332852 PMC1317329

[58] Bolger AM, Lohse M, Usadel B. 2014. Trimmomatic: a flexible trimmer for Illumina sequence data. Bioinformatics 30:2114–2120. 10.1093/bioinformatics/btu170. 24695404 PMC4103590

[59] Love MI, Huber W, Anders S. 2014. Moderated estimation of fold change and dispersion for RNA-seq data with DESeq2. Genome Biol 15:550. 10.1186/s13059-014-0550-8. 25516281 PMC4302049

[60] Varet H, Brillet-Guéguen L, Coppée J-Y, Dillies M-A. 2016. SARTools: a DESeq2- and EdgeR-based R pipeline for comprehensive differential analysis of RNA-Seq data. PLoS One 11:e0157022. 10.1371/journal.pone.0157022. 27280887 PMC4900645

[61] King ZA, Dräger A, Ebrahim A, Sonnenschein N, Lewis NE, Palsson BO. 2015. Escher: a Web application for building, sharing, and embedding data-rich visualizations of biological pathways. PLoS Comput Biol 11:e1004321. 10.1371/journal.pcbi.1004321. 26313928 PMC4552468

[62] Olivier BG. 2019. SystemsBioinformatics/cbmpy-metadraft: Metadraft. Zenodo. 10.5281/zenodo.2398336. Accessed 8 October 2019.

[63] Ebrahim A, Lerman JA, Palsson BO, Hyduke DR. 2013. COBRApy: COnstraints-Based Reconstruction and Analysis for Python. BMC Syst Biol 7:74. 10.1186/1752-0509-7-74. 23927696 PMC3751080

[64] Christensen JE, Steele JL. 2003. Impaired growth rates in milk of Lactobacillus helveticus peptidase mutants can be overcome by use of amino acid supplements. J Bacteriol 185:3297–306. 12754227 10.1128/JB.185.11.3297-3306.2003 PMC155375

[65] Richelieu M, Houlberg U, Nielsen JC. 1997. Determination of α-acetolactic acid and volatile compounds by headspace gas chromatography. J Dairy Sci 80:1918–1925. 10.3168/jds.S0022-0302(97)76132-0.

[66] Villas-Bôas SG, Smart KF, Sivakumaran S, Lane GA. 2011. Alkylation or silylation for analysis of amino and non-amino organic acids by GC-MS? Metabolites 1:3–20. 10.3390/metabo1010003. 24957242 PMC4012512

