# Supplementary material for: Genome-Scale Metabolic Modeling Combined with Transcriptome Profiling Provides Mechanistic Understanding of Streptococcus thermophilus CH8 Metabolism
Source: Appl Environ Microbiol. 2022 Aug 4;88(16):e00780-22. doi: 10.1128/aem.00780-22 (PMC9477255; doi:10.1128/aem.00780-22)

Supplemental Material:

- Figure S1: Clustering of *S. thermophilus* strains based on the presence or absence of predicted protein ortholog groups.
- File S1: *Streptococcus thermophilus* CH8 genome-scale metabolic model iRZ476.
- Table S1: Overview of *S. thermophilus* orthologs.
- Table S2: RNA-Seq sequencing statistics.
- Table S3: Table of differential expression and count levels for all genes.
- Table S4: Modeling data. Flux values and bounds of all reactions during growth on CDM with free amino acids, or with casein peptide as amino acid source. Also includes an overview of reaction curations in comparison to the existing *S. thermophilus* LMG18311 model.
- Table S5: Concentrations of free amino acids during milk fermentation

Figure S1: Clustering of *S. thermophilus* strains based on the presence or absence of predicted protein ortholog groups.

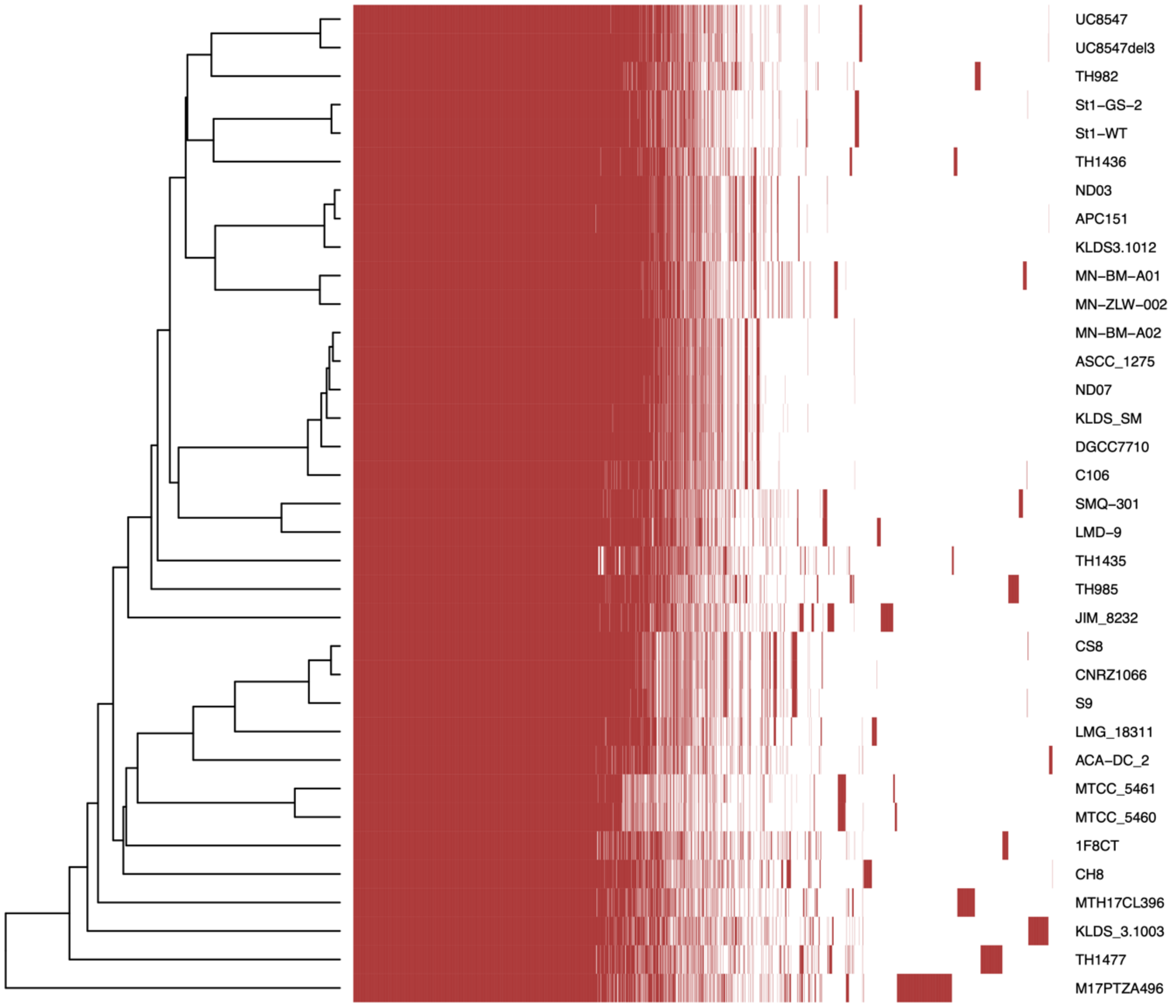

Supplement: Supplemental file 2 — Fig. S1 and descriptions of all supplemental material. Download aem.00780-22-s0007.pdf, PDF file, 0.3 MB [file aem.00780-22-s0007.pdf]
